# PA-12-Zirconia-Alumina-Cenospheres 3D Printed Composites: Accelerated Ageing and Role of the Sterilisation Process for Physicochemical Properties

**DOI:** 10.3390/polym14153152

**Published:** 2022-08-02

**Authors:** Damian S. Nakonieczny, Magdalena Antonowicz, Gražyna SimhaMartynkova, Frank Kern, Lenka Pazourková, Karol Erfurt, Michał Hüpsch

**Affiliations:** 1 Institute for Manufacturing Technologies of Ceramic Components and Composites, University of Stuttgart, 70569 Stuttgart, Germany; frank.kern@ifkb.uni-stuttgart.de; 2 Nanotechnology Centre, CEET, VŠB—Technical University of Ostrava, 17. Listopadu 15, 708733 Ostrava-Poruba, Czech Republic; grazyna.simha@vsb.cz; 3 Department of Biomedical Engineering, Silesian University of Technology, Akademicka 2A, 44-100 Gliwice, Poland; magdalena.antonowicz@polsl.pl (M.A.); michhup608@student.polsl.pl (M.H.); 4 IT4 Innovations, VŠB—Technical University of Ostrava, 17. Listopadu 15, 708733 Ostrava-Poruba, Czech Republic; lenka.pazourkova@vsb.cz; 5 Faculty of Chemistry, Department of Chemical Organic Technology and Petrochemistry, Silesian University of Technology, Krzywoustego 4, 44-100 Gliwice, Poland; karol.erfurt@polsl.pl

**Keywords:** polymer-ceramic composites, accelerated ageing tests, sterilisation, PA-12, zirconia, alumina, cenospheres

## Abstract

The aim of this study was to conduct artificial ageing tests on polymer-ceramic composites prepared from polyamide PA-12 polymer matrix for medical applications and three different variants of ceramic fillers: zirconia, alumina and cenospheres. Before ageing, the samples were subjected to ethyl oxide sterilization. The composite variants were prepared for 3D printing using the fused deposition modeling method. The control group consisted of unsterilized samples. Samples were subjected to artificial ageing in a high-pressure autoclave. Ageing conditions were calculated from the modified Hammerlich Arrhenius kinetic equation. Ageing was carried out in artificial saliva. After ageing the composites were subjected to mechanical (tensile strength, hardness, surface roughness) testing, chemical and structural (MS, FTIR) analysis, electron microscopy observations (SEM/EDS) and absorbability measurements.

## 1. Introduction

The field of biomaterials engineering continues to see an increased interest in composites, especially in dental and orthopaedic applications [1,2]. This is due to the fact that the use of composites in medicine solves many of the problems that affect metallic biomaterials including corrosion, susceptibility to microbial colonisation and human tissue-implant mechanical mismatch [3,4,5]. Polymer-ceramic composites (PCCs) have received particular attention due to their desirable physico-chemical properties such as abrasion resistance, the possibility of achieving a Young’s Modulus similar to that of human bones, aesthetic benefits, easy machinability and bioavailability [6,7]. PCCs are already used in restorative dentistry for repairing teeth with caries, while in dental prosthetics they are used for CAD/CAM milling blocks [8,9]. Additionally mobile restorations also use PCCs for prostheses and implant-supported screws [10,11]. With regard to orthopaedic applications, PCCs are utilised in osteosynthesis implants (i.e., plates, intramedullary nails and spinal implant components) [12,13]. An important issue when considering composites as biomaterials is to determine their resistance to degradation in body fluid environments. When using composites as biomaterials, attention should be paid to factors such as pH, physiologically relevant temperature (i.e., 37 °C), variable mechanical loads and interaction with biological compounds (i.e., proteins) [14,15]. It is important to note that the implanted material will remain in the body for more than 2 years, so there is a need to predict the exact behaviour of the material during this period in the body. Moreover, experimental data can be used to predict the behaviour of the implant material during long-term use. Hukins et al. reported several kinetic relationships that are an extension of the Hemmerlich equation resulting from the simplified protocol for accelerated ageing of medical devices [15,16]. Based on these equations, it is possible to approximately determine the reference temperature at which the effects begin and what appears to be the simplest practical solution. However, this approach only considers temperature as a factor in ageing and does not take into account other variables such as medical sterilisation of the material, fluid simulating body fluids or compounds simulating organic compounds (i.e., proteins, fats, etc.). Interesting algorithms for hostile environment, elevated temperature and cyclic deformation can be found in the report on polymer and polymer composites accelerated ageing and lifetime prediction techniques presented by Maxwell et al. [17]. Maxwell et al. reported ageing as a function of temperature over time takes the form of the equations of Arrhenius and Avrami [17]. The report contains numerous algorithm variants, most of which are modifications and expansions of the Arrhenius equation. However, the simplest and most appropriate equation appears to be a simple power equation similar to the Hemmerlich equation [16,17]. In the available data, however, there is a lack of correlation between the accelerated ageing tests and ageing protocols for PCC biomaterials, as is the case for zirconium oxide [18], and the environment in which ageing takes place.

The main aim of this study was to carry out accelerated ageing tests of PCCs consisting of ZrO_2_ and Al_2_O_3_, cenospheres (CSs) as fillers and PA-12 as a polymer matrix from which samples for degradation tests were prepared in the form of fused deposition modelling (FDM) 3D printing filaments. Polyamides have promising applications in 3D printing and composite preparation [19,20]. A second objective of the study was to determine the effect of the type of sterilisation and the medium used for accelerated ageing tests on the physicochemical properties of both types of PCCs. Samples were prepared for strength tests according to ISO 527:2012 [21]. Additionally, samples were divided into 2 groups: (I) unsterilised and (II) ethylene oxide (EO) sterilised. Artificial ageing tests were conducted in high-pressure autoclaves in the environment of artificial saliva under ageing conditions calculated from the Arrhenius kinetic equation modified by Hammerlich [15,16]. The mechanical properties (tensile strength, hardness and surface roughness), absorbability, surface topography and chemical composition of the PCCs as well as the chemical composition of the simulant solution were analysed after ageing. The motivation for conducting this study was to determine the effect of medical sterilization and artificial ageing on the composites we had previously prepared from PA-12. These issues are important because they allow us to assess the impact of the medical sterilization process commonly used for implants. An additional aspect of the research conducted is to predict the behavior of the implant material under artificial saliva conditions—and thus to predict the material’s behavior under long-term conditions. In earlier studies, we focused on a simple approach of the reachability of composites in a very simplified model with temperature. In this study, following our earlier experiments, we added a kinetic model to represent changes in human body conditions, which were not accurately represented in earlier tests.

## 2. Materials and Methods

### 2.1. Materials and Samples

#### 2.1.1. Ceramic Filler Modification

The samples were prepared as described in our previous studies [22,23]. Zirconia (ZRO-T6 IMERYS), alumina (Sumitomo, Sumicorundum AA-18) and 90 μm fraction of purified cenospheres (origin: GRES-2 Powerplant, Kazakhstan) were modified by a two-step process: (I) etching in piranha solution and (II) surface modification with APTES (3-aminopropyltriethoxysilane). The etching solution was prepared using H_2_SO_4_ (CAS: 7664-93-9, 95%, Acros Organics), and H_2_O_2_ (CAS: 7722-84-1, 30%, STANLAB) in a volumetric ratio of 3:1. The powders were poured together with Piranha solution into a round-bottomed flask and heated under constant reflux (100 °C, 15 min, continuous stirring at 350 rpm). Then, the ceramics were washed under vacuum with a water pump two times with 500 mL of deionized water. The pH of the ceramic slurries was neutralized with ammonia water (CAS 1336-21-6, 25%, AVANTOR). In the second stage, all ceramics were etched in APTES (3-aminopropyltriethoxysilane; CAS: 919-30-2, Acros Organics, A Cp10% solution of APTES in 2-propanol (PrOH) (CAS: 67-63-0, Acros Organics). Slurries were stirred on a magnetic stirrer (30 °C, 24 h, 250 rpm). After mixing, the suspension was exposed to ultrasound (*f* = 37 kHz, power 120%, 50 min, 30 °C, degas mode). All ceramics were filtered under reduced pressure with a water pump. Then, the powders were dried (forced air dryer, 12 h, 80 °C) and calcined in a muffle furnace (RENFERT Magma) at 450 °C in an air atmosphere (temperature gradient 9°/min, isothermal holding for 2 h, samples cooled together with the furnace).

#### 2.1.2. Filament Preparation

Polymer-ceramic filaments were prepared with a twin-screw extruder for the compounding and a single screw extruder for the filament preparation. To avoid hydrolysis, the PA-12 (VESTAMID PA12, Evonik) granulate was pre-dried at 50 °C for 10 h. Alumina, zirconia and CSs were dried at 150 °C for 10 h. The molecular weight of PA-12 ranged from 9100 to 16,600 g·mol^−1^. The EBVP 25/44D extruder from O.M.C. SRL (Saronno, Italy) was used for compounding. The ceramic powder and the polymer granules were dosed gravimetrically with a mass ratio: of 20% ceramic powder to 80% polymer (PA) for CSs and 30:70% for zirconia and alumina. The mass throughput setup was 4.2 kg/h at 100 rpm and 260 °C extruder temperature at the exit. Single-screw extruder from DR. COLLIN

GmbH (Ebersberg, Germany) was used for shaping with a 3 kg/h mass throughput at 14 rpm. After extrusion, the polymer ceramic melt was pulled with a pull-off force which depended on the crystallization degree of the carrier material. To set the pull-off force, filament diameters between 1.6 and 1.8 mm were required and recorded using a WIREMASTER and the ODAC 18 XY laser head from Zumbach (Orpund, Switzerland).

#### 2.1.3. Sample Preparation

**Figure 1:**
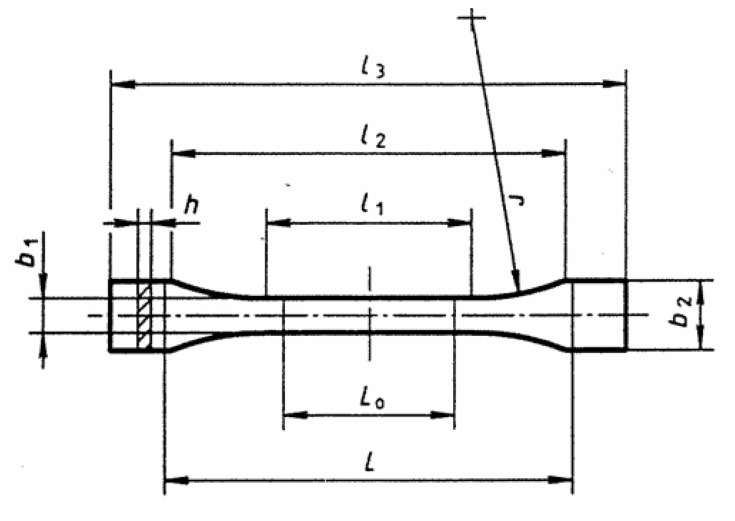
Sample type 1BA for strength test [21].

Sample preparation methods were analogous to those conducted in previous studies [21,22]. Samples were prepared by FDM printing on a 3D printer (Double P255 by 3D Gence, Gliwice, Poland). In accordance with the recommendations of ISO 527:2012 [A], type 1BA samples were prepared, which are preferred for machined specimens. The characteristic dimensions of the samples are shown in Figure 1 and Table 1. The 3D models were modelled in SolidWorks 2020 software (Dassault Systèmes SolidWorks Corporation, Waltham, MA, USA). In order to read the file by the printer, the file was saved in the STL format, and the appropriate printing parameters were selected using 3D Gence Slicer 4.0. software (3D Gence, Gliwice, Poland).

In the first stage, geometric samples for both materials were printed at extruder temperatures ranging from 200–220 °C to verify the quality of the surface and interlayer bonds. The printing parameters are shown in the Table 2. The printed samples were stored at 23 °C and 20% relative humidity.

The following table shows the designations and number of samples prepared for the experiments (Table 3). To compare repeatability in mechanical testing, five samples were prepared for each group. A total of 40 samples were prepared.

### 2.2. Sterilization

Sterilization was performed in ethylene oxide (EO) in an ANAPROLENE 74 sterilizer. Samples were inserted into standard sterilization sleeves made of paper. Sterilization was carried out based on the manufacturer’s standard program provided for polymers. After sterilization, the samples were aerated in the sleeves for 28 days in order to evaporate the residual EO.

### 2.3. Artificial Ageing Protocol

Artificial ageing was carried out according to the data and model recommendations of previous studies [15,16,17,24,25,26]. On this basis, the kinetic model of the Arrhenius equation transformed by Hammerlich was selected as the most suitable for our investigations:

(1) $$f=Q_{10}{}^{\frac{\Delta T}{10}}$$

where:

*f*—accelerated ageing factor (AAF),

*Q*_10_—conservative ageing factor, °C^−1^

Δ*T*—temperature variation, °C

where:

(2) $$\Delta T=T-T_{REF}$$

where:

*T_REF_*—reference temperature (representative for effects of ageing determination), °C

*T*—elevated temperature (used for accelerated ageing effects), °C

This equation is based on the empirical principle that increasing the temperature by about 10 °C roughly doubles the rate of many polymer reactions [15]. Therefore, the *Q*_10_ factor for this case will take the value of 2 when *T* = 10 °C and will equally take the form:

(3) $$f=2^{\frac{T-T_{REF}}{10}}$$

In addition, the artificial ageing time (*ATT*), which is related to the life test or real-time equivalent (*RTE*), must also be taken into account [17,26,27]:

(4) $$ATT=\frac{RTE}{f}$$

where:

*RTE*—days,

Based on the literature and data from ageing charts (equivalent to one year of room-temperature ageing plot), 1 year was selected as the *RTE* [15,25,26]. Thus, ageing parameters were determined for conditions in physiological fluids at elevated temperatures:

- simulated body solution (SBF)—artificial saliva (according to EN ISO 10993-15:2000, chemical composition, Table 4),
- *Q*_10_—2,
- *T*—97 °C,
- *T_REF_*—37 °C,
- *RTE*—365 days.

$$f=2^{\frac{\left(97-37\right)}{10}}$$

f=64

$$ATT=\frac{365}{64}$$

ATT=5.7 days

ATT=136 h 48 min

### 2.4. Artificial Ageing Process

Composite samples were kept sealed in high-pressure autoclaves (Carl ROTH, Model-1 without pressure gauge) and filled with artificial saliva (Table 4). Autoclaves were placed in a forced-air dryer (Binder FED) at 97 °C for 5.7 days. After the exposure time in artificial saliva, the samples were subjected to tests described below.

### 2.5. Mechanical Tests

The static tensile test was conducted in accordance with the recommendations of the standard EN ISO 527:2012 with a tensile speed of 5 mm/min, using the MTS Criterion Model 45 machine with a 10 kN force sensor and MTS TestSuite software (Eden Prairie, MN, USA). The separation between the grippers was 54 mm. Upon test completion, the maximum breaking force Fmax [N], the Young’s Modulus E [MPa], elongation at break A [%] and tensile strength Rm [MPa] were determined. Tests were carried out using a standard MTS extensometer. Five studies were performed for each type of sample. Measurements of microhardness were carried out using the Oliver and Pharr instrumental method, which is a measure of the resistance of a material to permanent deformation or damage and is defined as the quotient of the maximum applied loading force and the projected contact area between the indenter and the test sample [27]. The methodology was cross-referenced with other studies [28]. The tests were carried out using the open platform equipped with a Micro-Combi-Tester by CSM Instruments (Micro-Combi-Tester, CSM instruments a company of Anton Paar, Peseux, Switzerland) using a Vickers indenter. The micromechanical properties were determined on the basis of material deformation as a result of indentation of the sample with Vickers indenter to which a 100 mN maximal load was applied. The value of the loading force and the penetration depth of the indenter blade were recorded continuously during the entire cycle (loading and unloading). Loading and unloading rates were 200 mN/min with a 5 s hold time of the sample at maximum load. The value *f* the indenter load was resulting. The microhardness result is the average of 10 measurements measured longitudinally and transversely across the entire surface area. The values of indentation hardness (HIT) and Vickers hardness (HVIT) were determined.

### 2.6. FTIR—Fourier Transform Infrared

Tests were carried out using a Shimadzu IR Tracer-100 Fourier Transform Infrared Spectrophotometer (Michelson interferometer, beam splitter: KBr germanium coated, light source: high-energy ceramics, detector: DLATGS detector) using a multi-reflection ATR attachment equipped with a diamond prism. The device was calibrated with a closed ATR attachment, to record the background image. Then, the test samples were placed on the diamond and pressed against the prism with a dynamometric screw, each time with the same force. In order to analyze and interpret the characteristic bands of the tested samples, the transmission spectra were recorded on a multi-reflection device. The analysis was performed automatically using the dedicated LabSolution IR software (Shimadzu, Kioto, Japan) provided by the spectrometer manufacturer. To minimize error, 100 counts were performed with a resolution of 4 cm^−1^ for each analysis. All measurements were carried out in the medium infrared range of 4000–400 cm^−1^.

### 2.7. MS—Mass Spectrometry

The PA-12 standard was diluted in methanol and sonicated via ultrasound for 1 h to partially dissolve it in the solvent. The test samples were diluted with methanol and also sonicated with ultrasound for 1 h. These solutions were then examined by MS. High resolution MS analyses were performed on a Waters Xevo G2 Q-TOF mass spectrometer (Waters Corporation, Milford, MA, USA) equipped with an electrospray ionization (ESI) source operating in positive ion modes. MS data were collected from 100 to 2000 Da in positive ion mode with scan time of 0.1 s. To ensure accurate mass measurements, data were collected in centroid mode and mass was corrected during acquisition using leucine enkephalin solution as an external reference (Lock-SprayTM), which generated reference ions at m/z 556.2771 Da ([M + H]^+^) in positive ESI mode. The accurate mass and composition for the molecular ion adducts were calculated using the MassLynx software (Waters) incorporated with the instrument. Parameters and settings are presented below in Table 5. 9 samples were analyzed; 8 were samples aged in SBF and one was a blank sample of artificial saliva.

### 2.8. SEM/EDS—Scanning Electron Microscopy/Energy Dispersive Spectroscopy

Morphology observations of samples were made using a TESCAN VEGA scanning electron microscope (SEM) equipped with secondary electrons (SE) and backscattered electrons (BSE) detectors in low vacuum. Chemical composition was determined using energy dispersive X-ray spectroscopy (EDS, Oxford Instruments EDS probe with Aztec software).

### 2.9. Saturation Test

By applying the method used to determine the absorbency of polymers exposed to boiling water, the absorbency of the composite samples was determined. After artificial ageing, samples were weighed on an analytical balance with an accuracy of ±1 mg and then dried in a forced air circulation dryer (Binder FED) for 24 h at 50 °C. The absorbability of the prepared composites was then calculated from the equation below:

(5) $$A=\frac{m_{2}-m_{1}}{m_{2}}\cdot \;100\%$$

where:

*A*—absorbability, %

*m*_2_—sample weight after ageing, g

*m*_1_—sample weight after drying, g

**Figure 2:**
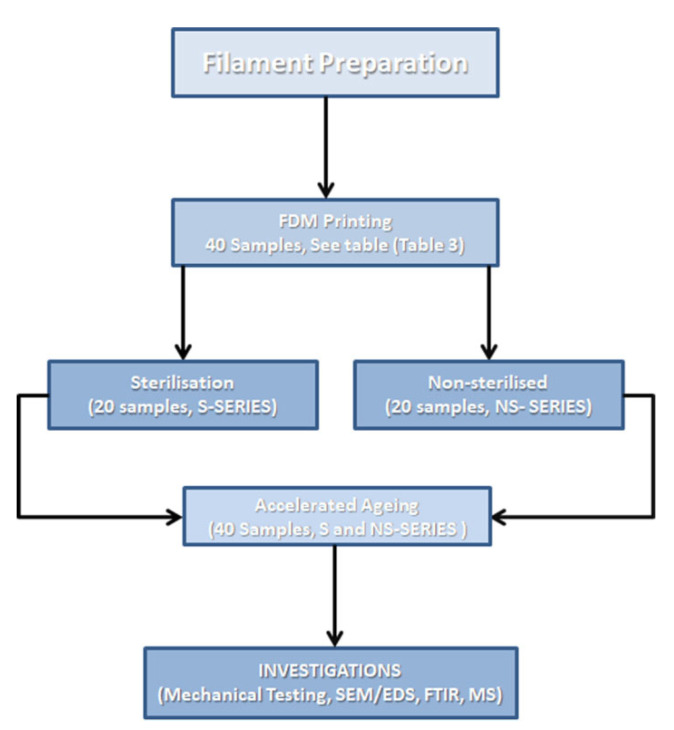
Process flow chart.

The entire study was designed as follows: the samples were divided into two main groups: non-sterilized (NS) 20 samples, and sterilized (S) also 20 samples (Table 3). On this basis, the effect of sterilization was assessed. With regard to sterilization, all 40 samples were subjected to the ageing protocol outlined above. This approach made it possible to determine exactly what the impact of sterilization and artificial ageing is. The process flow-chart with sample description below shows the extent of sample preparation and investigations (Figure 2). The process flow diagram is complementary to the table (see Table 3).

## 3. Results and Discussion

### 3.1. Results

#### 3.1.1. Mechanical Testing

##### Microhardness

Microhardness test results for all samples are presented in Table 6. Measurements were taken in different areas of the samples and no differences were found across the entire area.

##### Tensile Test

**Figure 3:**
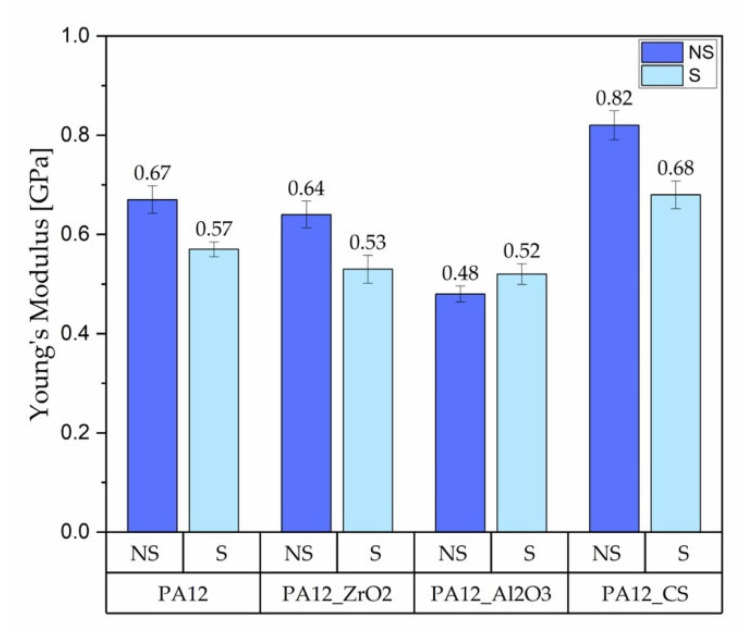
Young’s Modulus of the PA12, PA12_ZrO_2_, PA12_Al_2_O_3_ and PA12_CS samples in their non-sterilised/initial state and after sterilisation.

**Figure 4:**
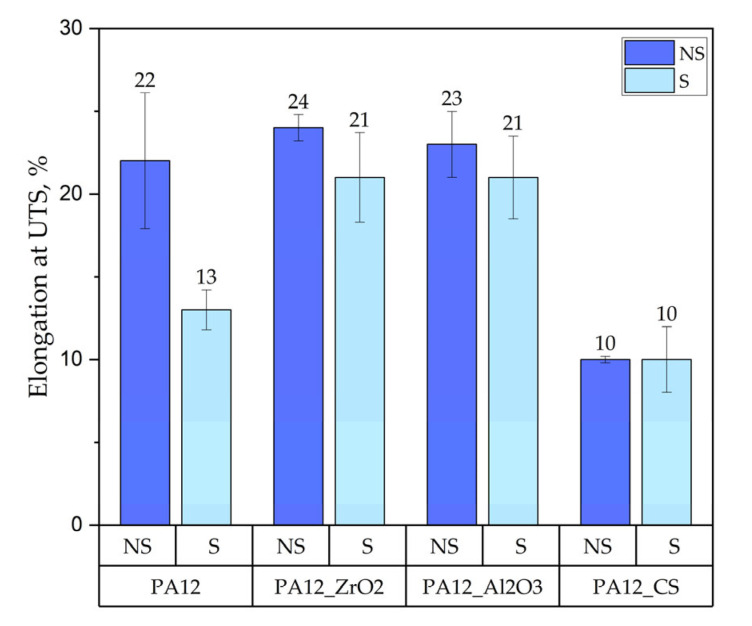
Elongation at ultimate tensile strength of the PA12, PA12_ZrO_2_, PA12_Al_2_O_3_ and PA12_CS samples in their non-sterilised/initial state and after sterilisation.

**Figure 5:**
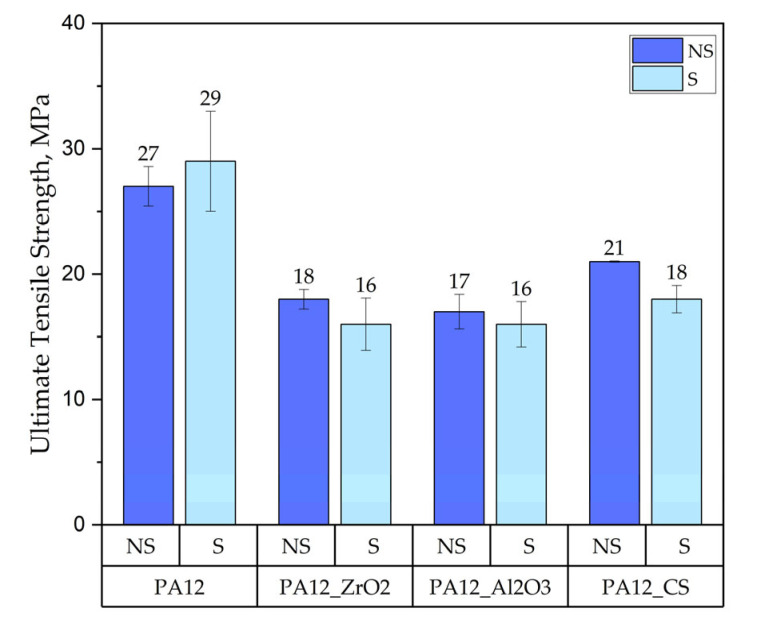
Ultimate tensile strength of the PA12, PA12_ZrO_2_, PA12_Al_2_O_3_ and PA12_CS samples in their non-sterilised/in initial state and after sterilisation.

The tensile test results of the specimens PA12, PA12_ZrO_2_, PA12_Al_2_O_3_ and PA12_CS in their initial/non-sterilised (NS) state and after sterilisation (S) are shown in Figure 3, Figure 4 and Figure 5.

#### 3.1.2. FTIR

**Figure 6:**
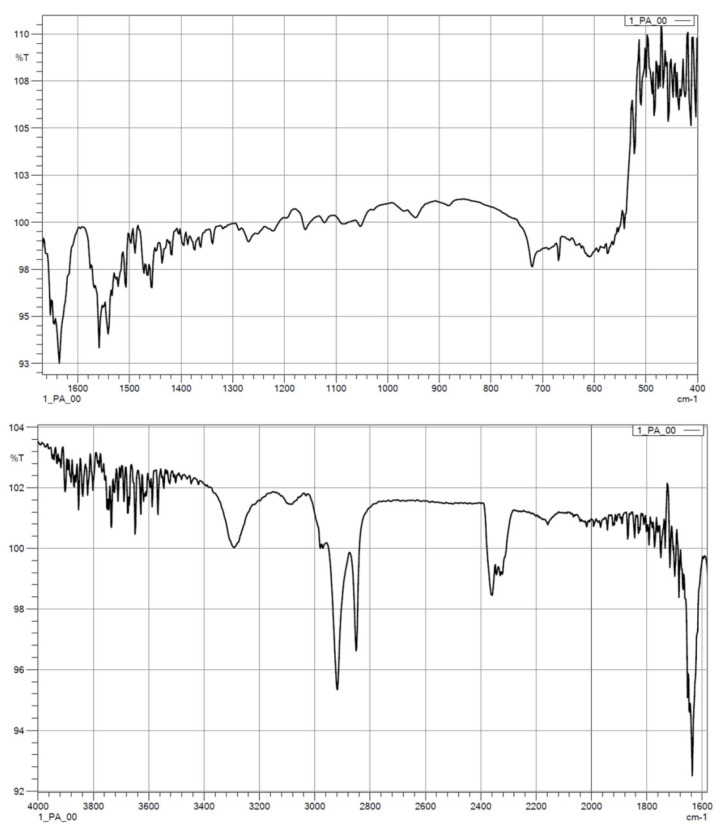
IR spectra for pure PA-12 before artificial ageing.

**Figure 7:**
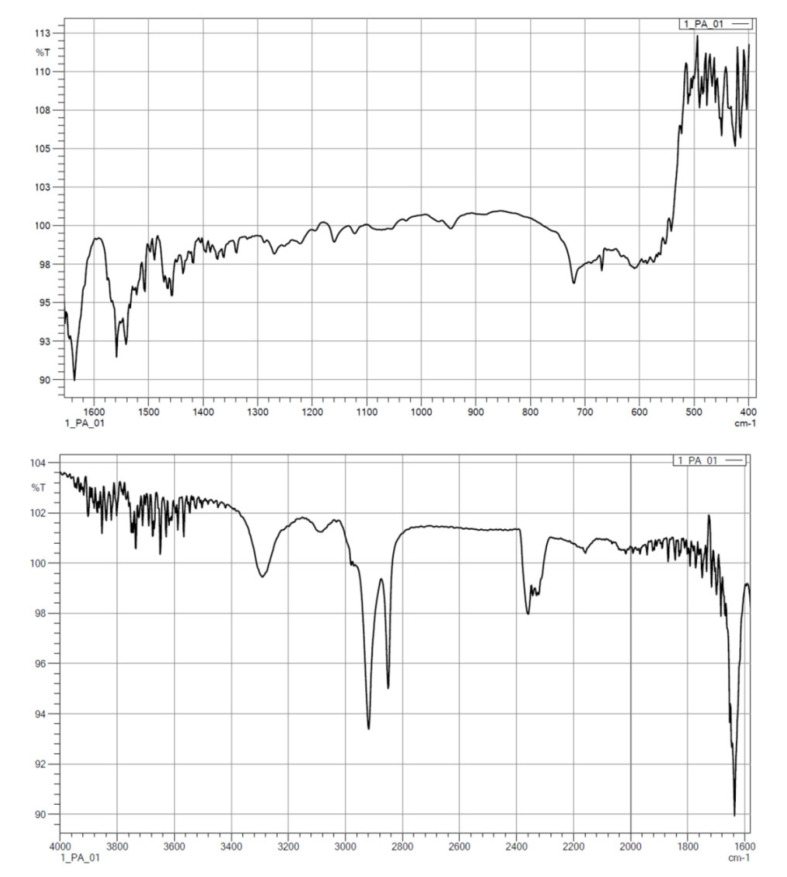
IR spectra for pure PA-12 after artificial ageing.

**Figure 8:**
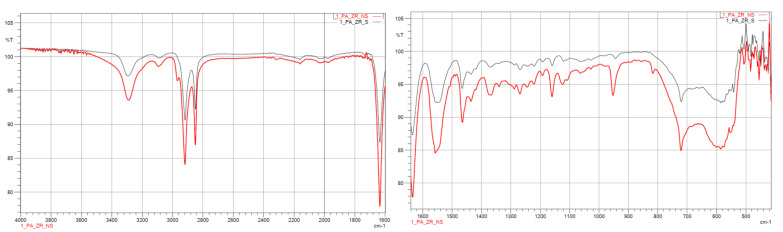
IR spectra for PA12_ZrO_2_ non-sterilised (red curves) and sterilised (black curves) samples.

**Figure 9:**
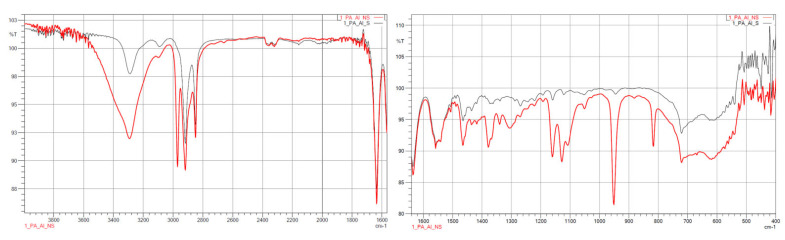
IR spectra for PA12_ Al_2_O_3_ non-sterilised (red curves) and sterilised (black curves) samples.

**Figure 10:**
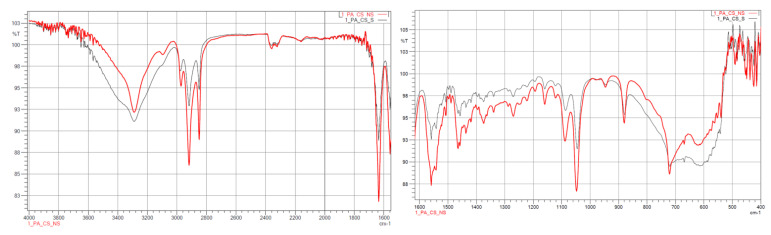
IR spectra for PA12_CS non-sterilised (red curves) and sterilised (black curves) samples.

The results from the IR measurements are presented below (Figure 6, Figure 7, Figure 8, Figure 9 and Figure 10). The identified characteristic bands are presented in Table 7 and Table 8 for pure PA-12 and samples with ceramic fillers, respectively [29,30]. The IR pattern of pure PA12 before and after artificial ageing are found in Figure 6 and Figure 7. For these graphs, the results from the IR analysis are shown in the table (Table 7). No significant differences in the IR spectrum were observed for PA-12 after artificial ageing compared to the non-sterilised state (Figure 6 and Figure 7, Table 7). A similar effect was observed for samples with ceramic fillers, but there were visible effects of surface modification with nitrogen and silicon compounds, which originate from APTES [29,30,31]. Detailed results for these samples are shown in the table (Table 8).

#### 3.1.3. MS

**Figure 11:**
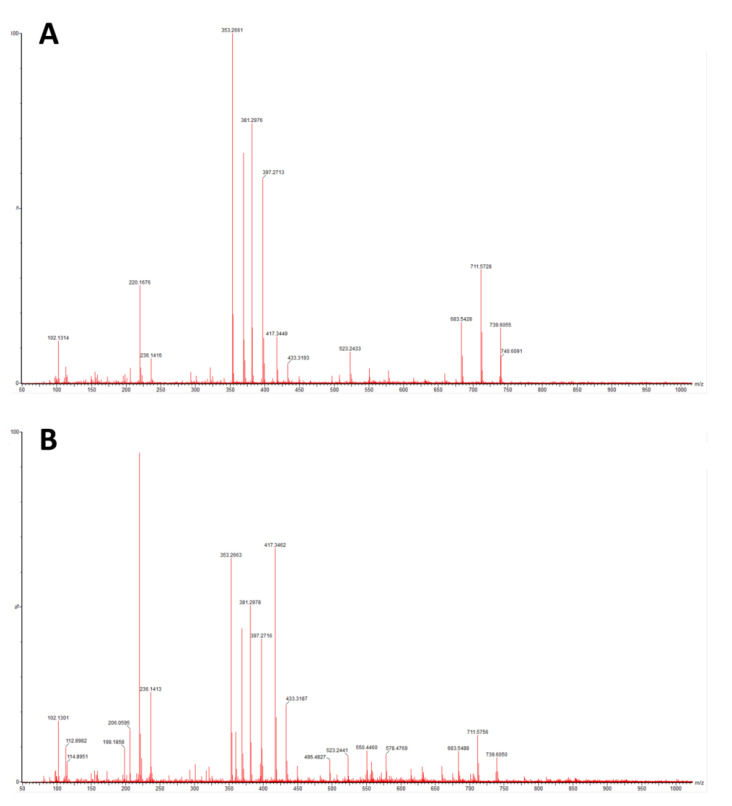
MS spectra for (**A**) PA12_CS_NS and (**B**) PA12_CS_S samples.

The prepared PA-12 standard produced signals for a laurolactam dimer with mass 395.3637 Da and sum formula C_24_H_46_N_2_O_2_ and a laurolactam trimer with mass 592.5417 Da and formula C_36_H_69_N_3_O_3_. No PA-12-derived signals were identified in the samples tested. Below selected representative results for PA12_CS_NS and PA12_CS_S samples are presented. The spectrum from the MS is shown in Figure (Figure 11).

#### 3.1.4. SEM/EDS

In PA12 samples, the break area appeared elastic and tractile, with huge dents containing irregular shapes and the creation of pulled-out ribbons. Non-sterilised and sterilised PA12 samples appeared very similar.

The break area of PA12_ZrO_2_ samples exhibited a fine porous morphology. There were many small particles of fillers and some larger agglomerates. Generally, the sterilised samples were more compact.

The break area of PA12_Al_2_O_3_ samples exhibited a combination of fine porous morphology and larger elongated pores. Additionally, visible fibrous particles of polymer and many regular particles of ceramic fillers, larger than those of zirconia, were also present.

The break area of PA12_CS samples had a similar pore morphology as Al_2_O_3_ samples. Pores were larger than 500 μm. Filler particles were randomly dispersed throughout the matrix as well as the appearance of fibrous polymer particles.

#### 3.1.5. Saturation Test

Below are the results from the saturation tests, which were measured on an analytical balance. The results are given using the u-measurement from the conversion (Equation (5)) in Table 9. The table shows the result from the soaking tests (Table 10).

### 3.2. Disscussion

The main objective of this study was to determine the effect of artificial ageing in artificial saliva and role of the medical sterilization for PCC composites with PA-12, ZrO_2_, Al_2_O_3_ and CS. The research builds on and continues the work prepared earlier [22,32]. In this study, we focused on the role of artificial ageing and sterilization on previously prepared PCC. Earlier studies presented PCC studies without ageing. Therefore, the kinetic model according to Arrhenius transformed by Hammerlich and EO sterilization were chosen [15,16,17]. This model was chosen as one of the few models which incorporate ageing time and temperature. Therefore, in our work we hypothesized that this would be the initial mathematical model for further investigation of composite biomaterial degradation.

**Figure 12:**
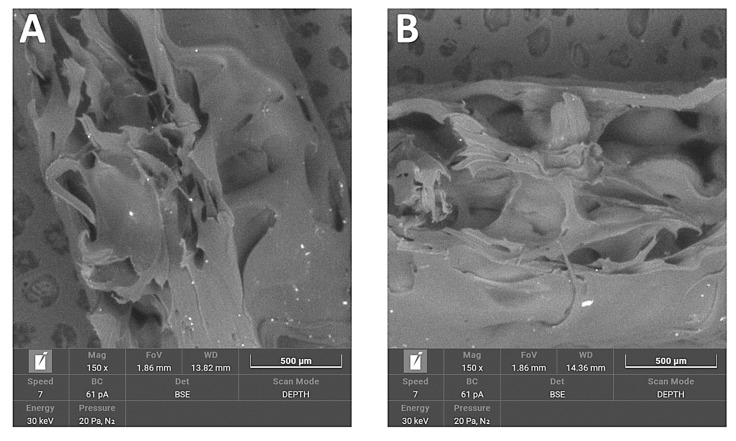
SEM micrographs used to compare breakthroughs of (**A**) PA12_NS and (**B**) PA12_S samples.

**Figure 13:**
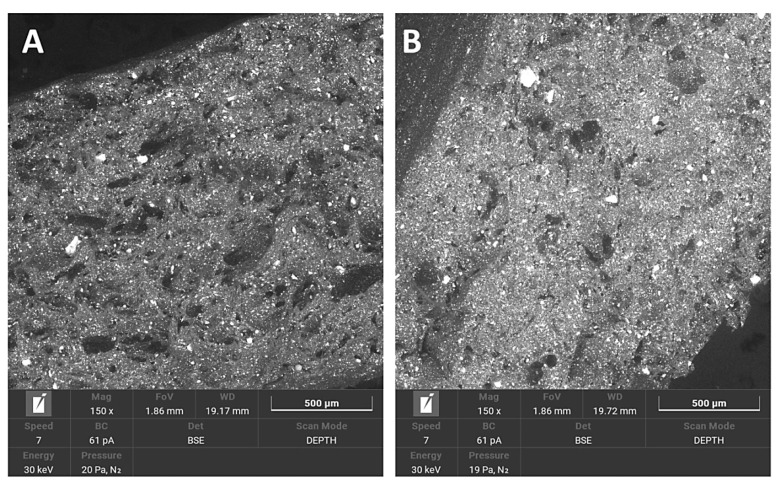
SEM micrographs used to compare breakthroughs of (**A**) PA12_ZrO_2__NS and (**B**) PA12_ZrO_2__S samples.

**Figure 14:**
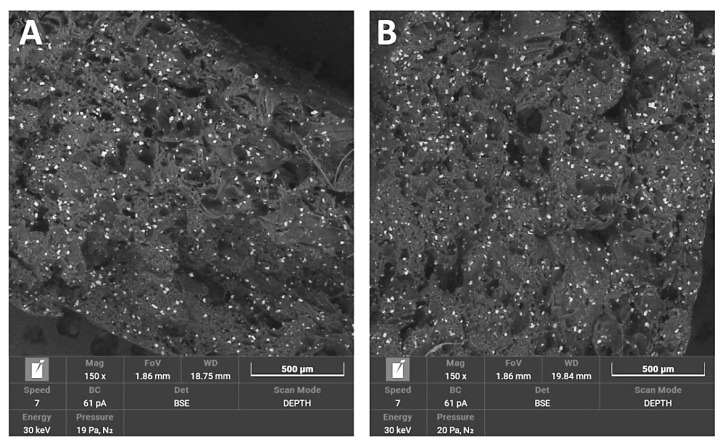
SEM micrographs used to compare breakthroughs of (**A**) PA12_ Al_2_O_3__NS and (**B**) PA12_ Al_2_O_3__S samples.

**Figure 15:**
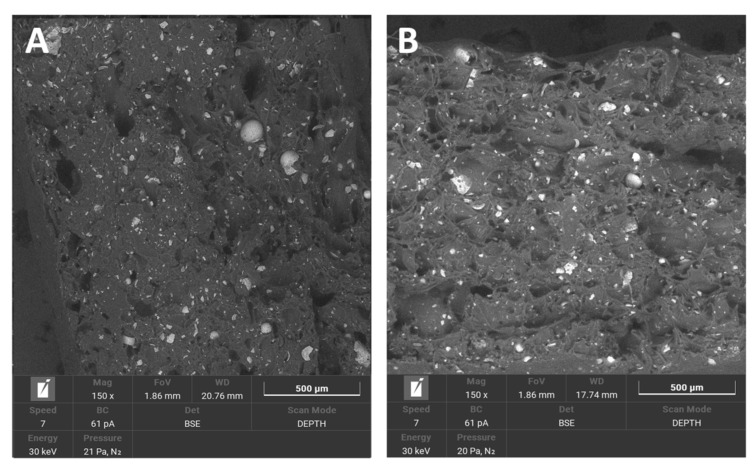
SEM micrographs used to compare breakthroughs of (**A**) PA12_CS _NS and (**B**) PA12_CS_S samples.

Regarding mechanical properties, a few differences were noted compared to our previous study [22]. For the starting state of pure PA-12 the E-modulus (E, Young’s Modulus) is 0.67 GPa (unsterilized) and 1.05 GPa in the earlier study [22]. However, regarding the starting states for PA12_Al2O3 and PA12_ZrO_2_, 0.62 GPa for PA-12 alumina reinforced and 0.63 GPa for zirconia reinforced composites were recorded in the earlier study [22]. In contrast, Young’s Modulus values were 0.48 and 0.64 for PA12_Al_2_O_3_ and PA12_ ZrO_2_ samples, respectively, in this study. Drying conditions of the PA-12 granulate could be responsible for the differences in Young’s Modulus. Partial hydrolysis that takes place in the granulate has a negative effect on the E-modulus, UTS (ultimate tensile strength) and elongation changed significantly. This can be seen, for example, in the elongation of the samples. In earlier studies, elongation was 9 % for PA12_ ZrO_2_ and 14% PA12_Al_2_O_3_. In this study the elongation percentages were 24 and 23% for PA12_ ZrO_2_ and PA12_Al_2_O_3_ samples, respectively [22]. Another possible reason for the discrepancies of the mechanical properties in this study compared to previous studies is the moisture content of the zirconia and alumina fillers. For PA12_CS samples, there were no such low values for E-modulus, UTS and elongation (Figure 3, Figure 4 and Figure 5). Residual moisture can significantly worsen the mechanical properties of the samples. Hence, it can be concluded that this sample preparation error negatively affected the properties of the overall composite. However, the effect of sterilization impact on the mechanical properties is clearly discernible. Almost all samples were negatively impacted by EO sterilization (Figure 3, Figure 4 and Figure 5). In most samples the Young’s Modulus decreased to about 20% (Figure 3). In contrast, the modulus of PA12_Al_2_O_3__S samples increased ~7.7%. The obtained results seem to confirm the observations made by Horakova et al. while other authors studied the effect of EO sterilization on PCL (polycaprolactone) [33]. They noted that using EO for sterilization compared to the non-sterilized samples, the EO sterilization cycle lowered the elastic properties. These effects were also observed for PA-12 samples (Figure 4). At the same time, Horakova et al., found that the effect of sterilization on E-modulus and UTS is less observable [33]. In general, in each group of samples, EO sterilization resulted in an increase of degradability in SBF; the only deviation was observed for pure PA-12 samples at UTS. However, as far as the results of HV hardness and HIT microhardness measurements are concerned, the results are very divergent (Table 6). For pure PA-12 samples, EO sterilization had a negative effect causing a decrease in hardness. For composites with zirconia and alumina, a significant increase in microhardness was observed. The microhardness of PA12_Al_2_O_3__S samples increased 13.6%, and PA12_ZrO_2_ samples increased about 74%. These phenomena can be justified by the fact that PA-12 is a semi-crystalline thermoplastic and the degradation conditions used resulted in an increase in its crystallinity [34]. These conclusions are confirmed by SEM observations, where long drawn-out PA-12 fibers can be seen in the fractures of unsterilized samples. By contrast, in sterilized samples the fractures are sharp-edged (Figure 12Figure 13, Figure 14 and Figure 15).

With regard to trying to determine the effect of accelerated ageing on the prepared PCC’s, FTIR and mechanical tests were chosen. From all spectra, no negative effect of artificial ageing in SBF was found. For pure PA-12 before and after ageing (spectra in Figure 5 and Figure 6), the results are shown in Table 7. And for PA-12 with ceramic fillers (Figure 7, Figure 8 and Figure 9), the results from the spectra are shown in Table 8. No functional groups that could originate from artificial saliva were identified, particularly phosphates and chlorides (Table 4). Only the presence of nitrogen and silicon compounds from the APTES surface modification process was confirmed, which we have already observed in previous studies [23]. The observations from FTIR were related to the results from the EDS, which are shown in the table (Table 8). No significant differences were observed for any of the samples. The effect of surface modification for all types of fillers (presence of N atoms) is well visible. On the other hand, a trace of the degradation process is apparent due to the presence of K and Cl ions, which may originate from KSCN or KCl. However, no correlation can be stated with certainty as to which types of materials have a higher affinity for absorbing compounds from SBF. The observations for FTIR were also correlated with the results from MS (Figure 11A,B). No evidence of PA-12 degradation in SBF was found in the tested artificial saliva samples after ageing.

A comparison of the effect of ageing on mechanical properties was performed for samples from earlier studies [22,32] and samples from this study that had been aged but not sterilized (NS_Series). We obtained the following results:

- for composites with ZrO_2_, for composites without ageing the values were E = 0.63 GPa, UTS = 11 MPa, elongation 9 %, HVIT = 11, HIT, = 120 MPa; while for composites after ageing: E = 0.64 GPa, UTS = 18 MPa, elongation 24 %, HVIT = 9, HIT, = 100 MPa;
- for composites with Al_2_O_3_, for composites without ageing the values were E = 0.62 GPa, UTS = 13 MPa, elongation 14 %, HVIT = 17, HIT, = 175 MPa; while for composites after ageing: E = 0.48 GPa, UTS = 17 MPa, elongation 23 %, HVIT = 15, HIT, = 125 MPa;
- for composites with CS, for composites without ageing the values were E = 0.82 GPa, UTS = 21 MPa, elongation 10 %, HVIT = 29, HIT, = 302 MPa; while for composites after ageing: E = 0.68 GPa, UTS = 21 MPa, elongation 10 %, HVIT = 27, HIT, = 290 MPa;

Hence, it can be concluded that the composite with CS is the most susceptible to changes in properties after ageing. Of all the composite variants, it is interesting to note that PA_12_CS did not change elongation in tension—for the others, a clear increase in ductility is apparent. This is most likely related to the volume of the ceramic filler—with an increase in volume, a decrease in the composite’s plasticity and a decrease in the value of Young’s Modulus is observed. The analysis of the effect of ageing shows that, in general, E-modulus and hardness decrease and the composite becomes more ductile (PA_12_CS is different due to the significant volume share of CS, making the composite stiffer).

**Figure 16:**
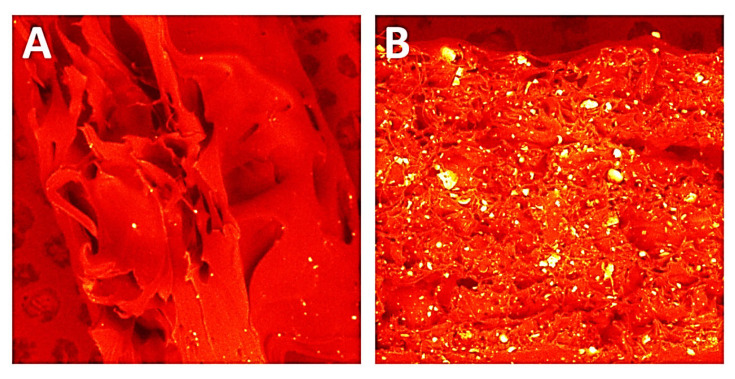
Dispersion of ceramic fillers in the polymer matrix for (**A**) PA12_NS and (**B**) PA12_CS_S samples. Visible impurities from the zirconia injection moulding process were found in PA12 samples.

**Figure 17:**
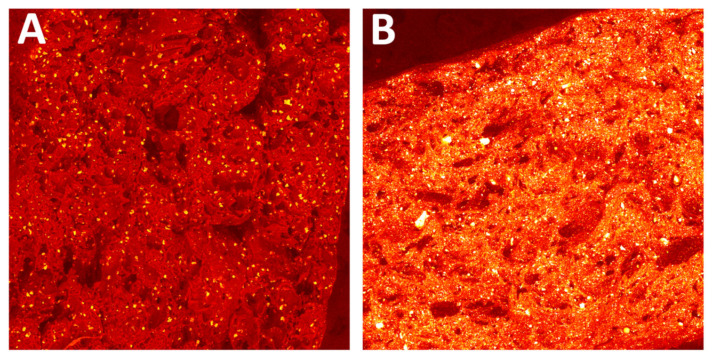
Dispersion of ceramic fillers in the polymer matrix for (**A**) PA12_Al_2_O_3__S and (**B**) PA12_ZrO_2_NS samples.

Morphology changes were observed in SEM micrographs (Figure 12, Figure 13, Figure 14 and Figure 15), which correlated with the results from the mechanical tests. In unsterilized EO composites, elongated PA-12 fibers at the breakthrough were observed proving higher ductility of NS samples in comparison with S samples. This observation was most apparent for PA12_Al2O3_NS samples in Figure 14A. In contrast, these differences were not observed for composites with CSs (Figure 15). This can be explained by the fact that CSs have a higher degree of packing in the polymer matrix and more random dispersion than zirconia and alumina, which is also evident from the dispersion comparison images (Figure 16 and Figure 17). This observation correlates with our observations from the granulate preparation process for the filament. CSs agglomerate and do not mix very well in the extruder with PA-12. In our earlier work we pointed out that the maximum weight percent we could add was 20% CS to 80% PA-12 [32]. Therefore, this could also be the direct reason for this behavior of the PA12_CS_NS/S samples. We can guess that this is mainly due to problems with wetting of the CS surface by the liquid polymer and poor adhesion of the ceramic to the polymer.

With regard to water absorption, we were not surprised by the results. Pure PA-12 proved to be the most absorbent, followed by PA12_Al_2_O_3_, PA12_ZrO_2_, and then PA12_CS samples. The absorbability is mainly a function of the density of the ceramic filler. An increase in filler density increases the absorbability (inversely to its volume). The densities of the ceramic fillers are 5.70 g·cm^−3^, 3.95 g·cm^−3^, and 0.84 g·cm^−3^ for ZrO_2_ [35], Al_2_O_3_ [36], and CS [32], respectively. Touris et al. report an absorbability value of 0.693% for the PA-12 catheter jacket composed of Grilamid L25 (PA-12 by EMS-Grivory) after 48 h of soaking [37]. Here, we report an absorbability value of 5.71% for PA-12. This can be explained by improper drying of PA-12 before granulate preparation and the presence of residual moisture in bulk PA-12.

## 4. Conclusions

In this study, we evaluated the effect of artificial ageing in SBF for PA-12 composites with zirconia, alumina and CSs. The ageing model was calculated according to Hammerlich’s modified Arrhenius kinetic equation. The artificial ageing test was calculated so that the entire degradation process corresponds approximately to the exposure of human saliva at 37 °C for 365 days. The following conclusions were drawn from conducted investigations:

1. in all of the prepared composites, changes in the values of mechanical properties
2. as a result of artificial ageing have been observed; in comparison to earlier studies [22,32], a decrease in the value of the Young’s Modulus as well as compression set and UTS have been observed; in contrast, the UTS of non-sterilised PA-12 samples increased after ageing; it is difficult to discern any correlation between the ageing process and changes in microhardness and hardness due to large variability; therefore, further studies are required;
3. EO sterilisation tended to cause deterioration of mechanical properties. However, in the case of hardness changes there is a large variability, hence further research is needed on this topic;
4. slight interaction with artificial saliva was found during ageing in PA-12 and composite samples as evidenced by the presence of K and Cl ions observed using EDS;
5. artificial saliva testing with MS after ageing did not reveal the presence of PA-12 breakdown products or any composite components;
6. in the case of PA-12 with ceramic fillers, the absorbability is a function of the filler density. The sample absorbability is greater with increased density and decreased volume.

## Figures and Tables

**Table 1:** Sample dimensions.

Dimensions of the Sample	Dimensions, mm
*l*_3_—overall length	75
*l*_1_—length of narrow parallel-sided portion	30.5
*r*—radius	37
*l*_2_—distance between broad parallel-sided portions	57.5
*b*_2_—width at ends	10
*b*_1_—width of narrow portion	5
*h*—thickness	2.35
*L*_0_—gauge length	25
*L*—initial distance between grips	54

**Table 2:** Processing parameters of the soaking test samples.

Printing Parameters		
Material	PA-ZrO_2_	PA-Al_2_O_3_
Nozzle diameter (mm)	0.5 mm	0.5 mm
Layer thickness (mm)	0.35 mm	0.35 mm
Build orientation	× (horizontally)	× (horizontally)
Infill density	100%	100%
Infill pattern	Linear aligned (0°)	Linear aligned (0°)
Outer layers	2	2
Extruder temp. (°C)	210 °C	210 °C

**Table 3:** Sample description.

Samples	Description	Non-Sterilised Samples	Sterilised Samples
PA12	Pure polyamide	PA12_NS (5 samples)	PA12_S (5 samples)
PA12_ZrO_2_	Polyamide and zirconia	PA12_ZrO_2__NS (5 samples)	PA12_ZrO_2__S (5 samples)
PA12_Al_2_O_3_	Polyamide and alumina	PA12_Al_2_O_3__NS (5 samples)	PA12_Al_2_O_3__S (5 samples)
PA12_CS	Polyamide and cenosphere	PA12_CS_NS (5 samples)	PA12_CS_S (5 samples)

**Table 4:** *SBF*—Artificial saliva chemical composition.

Compound	Na_2_HPO_4_	NaCl	KSCN	KH_2_PO_4_	NaHCO_3_	KCl
**Concentration, g/L**	0.260	0.700	0.330	0.200	1.500	1.200

**Table 5:** MS parameters.

Settings	Characteristics
Polarity:	ES+
Analyzer:	Resolution Mode
Capillary (kV):	40,000
Sampling Cone:	200,000
Extraction Cone:	40,000
Source Temperature (°C):	120
Desolvation Temperature (°C):	200
Cone Gas Flow (L/Hr):	50.0
Desolvation Gas Flow (L/Hr):	500.0

**Table 6:** Microhardness results of tested samples.

	PA12_NS	PA12_S	PA12 ZrO_2__NS	PA12 ZrO_2__S	PA12 Al_2_O_3__NS	PA12 Al_2_O_3__S	PA12 CS_NS	PA12 CS_S
Vickers hardness, *HV_IT_*	12 ± 2	11 ± 2	9 ± 2	16 ± 9	15 ± 1	13 ± 4	27 ± 2	22 ± 4
Microhardness *H_IT_,* MPa	122 ± 19	113 ± 9	100 ± 20	174 ± 18	125 ± 9	142 ± 31	290 ± 7	240 ±10

**Table 7:** IR band description for PA-12 samples.

Wave Number, cm^−1^	Characteristics
1500	C=O
1539–1645	N–H, C–CO–NH_2_, C=O
1540	–NH
1650	–CO
2800	alkyl groups
2900	alkyl groups
3500	–OH
3750	free–NH

**Table 8:** IR band description for PA12_ZrO_2_, PA12_Al_2_O_3_, and PA12_CS samples.

Samples	Wave Number, cm^−1^	Characteristics
PA12_ZrO_2__NS/S PA12_Al_2_O_3__NS/S PA12_CS_NS/S	460	Si–O–Si
	1000 ÷ 1100	Si–O
	1484	–NH_2_
	1500	C=O
	1539 ÷ 1645	N–H, C–CO–NH_2_, C=O
	1540	–NH
	1562	–NH_2_
	1650	–CO
	2800	alkyl groups
	2900	alkyl groups
	3500	–OH
	3750	free–NH

**Table 9:** Comparison of the chemical composition from EDS investigations: samples before and after artificial ageing.

Sample	Chemical Composition, wt%												
	C	O	Al	Zr	N	Fe	Na	K	Mg	P	Si	S	Cl
PA12 *	84.7	15.3	-	-	-	-	-	-	-	-	-	-	-
PA12_NS/S	81.8	-	-	-	-	-	1.2	2	-	-	-	-	-
PA12_ZrO_2_ *	78.7	14.4	-	3.6	3.3	-	-	-	-	-	-	-	-
PA12_ZrO_2__NS/S	76.0	15.1	-	3.5	3.2	-	0.5	0.7	-	0.3	-	-	0.7
PA12_Al_2_O_3_ *	75.1	20.1	3.0	-	1.8	-	-	-	-	-	-	-	-
PA12_Al_2_O_3__NS/S	75.2	17.2	3.1	-	1.6	-	1.0	0.9	-	0.2	-	-	0.8
PA12_CS *	78.7	14.4	0.8	-	3.3	0.3	-	-	1.2	-	1.3	-	-
PA12_CS_NS/S	75.0	15.1	0.7	-	3.8	0.3	0.8	0.6	1.1	0.5	1.1	-	1.0

* Samples that did not undergo the ageing process.

**Table 10:** Results of soaking tests.

Sample	Mass before the Absorption Measurement, g	Mass after Measurement of Absorption, g	Absorbability, % *
PA12	0.710	0.753	5.71
PA12_ZrO_2_	0.854	0.867	1.50
PA12_Al_2_O_3_	0.979	1.001	2.20
PA12_CS	0.793	0.801	1.00

* Results were rounded to two significant digits.

## Data Availability

Not applicable.
